# Tunneling a crosstown highway: a natural experiment testing the longitudinal effect on physical activity and active transport

**DOI:** 10.1186/s12966-021-01180-1

**Published:** 2021-08-26

**Authors:** Nicole E. H. Stappers, Jasper Schipperijn, Stef P. J. Kremers, Marleen P. M. Bekker, Maria W. J. Jansen, Nanne K. de Vries, Dave H. H. Van Kann

**Affiliations:** 1 Department of Health Promotion, Maastricht University, NUTRIM School of Nutrition and Translational Research in Metabolism, P. Debyeplein 1, 6229HA, Maastricht, The Netherlands; 2 Research Unit for Active Living, Department of Sports Science and Clinical Biomechanics, University of Southern Denmark, Odense, Denmark; 3 Department of Health Services Research, Maastricht University, CAPHRI Care and Public Health Research Institute, Maastricht, The Netherlands; 4 Center for Space, Place and Society, Social Sciences Group, Wageningen University, Wageningen, The Netherlands; 5 Academic Collaborative Center for Public Health, Public Health Service South-Limburg, Heerlen, The Netherlands; 6 Department of Health Promotion, Maastricht University, CAPHRI Care and Public Health Research Institute, Maastricht, The Netherlands; 7 Fontys University of Applied Sciences, School of Sport Studies, Eindhoven, The Netherlands

**Keywords:** Infrastructural change, built environment, physical activity, active transport, global positioning systems (GPS)

## Abstract

**Background:**

In the city of Maastricht in the Netherlands, a highway crossing several deprived neighborhoods was tunneled in 2016. The vacant space on top of this tunnel was redesigned and prioritized for pedestrians and cyclists. The aim of this study was to evaluate the effect of this major infrastructural change, named the Green Carpet, on total and transport-based physical activity (PA) levels.

**Methods:**

Participants (≥18 years) were part of one of three area-based exposure groups. The maximal exposure group lived in neighborhoods directly bordering the Green Carpet. The minimal exposure group consisted of individuals living at the other side of the city, and the no exposure group consisted of individuals living in a nearby city. Actual use of the new infrastructure was incorporated as a second measure of exposure. Data were collected before and 3-15 months after the opening of the Green Carpet. Device-based measurements were conducted to obtain PA levels and collect location data. Changes in PA over time and intervention effects were determined using linear mixed models.

**Results:**

PA levels in the Green Carpet area increased for the maximal and minimal exposure groups, but did not lead to an increase in total or transport-based PA. For the no exposure group, transport-based MVPA decreased and transport-based SB increased. The significant interaction (time x exposure) for transport-based SB, indicated differences in trends between the no exposure and maximal exposure group (B=-3.59, 95% CI - 7.15; -0.02) and minimal exposure group (B= -4.02, 95% CI -7.85, -0.19). Trends in the results based on analyses focusing on actual use and non-use of the new infrastructure were similar to those of the area-based analyses.

**Conclusions:**

Results suggest that the Green Carpet led to more PA in this specific area, but did not increase the total volume of PA. The area-based differences might reflect the differences between users and non-users, but we should be careful when interpreting these results, due to possible interference of selective mobility bias. This paper reflects that the relationship between infrastructure and PA is not unambiguous.

**Trial registration:**

This research was retrospectively registered at the Netherlands Trial Register (NL8108).

**Supplementary Information:**

The online version contains supplementary material available at 10.1186/s12966-021-01180-1.

## Background

The detrimental effects of physical inactivity on non-communicable diseases have been widely studied and the results highlight the need to increase population-wide physical activity (PA) levels in order to improve public health and decrease healthcare costs [1, 2]. According to socioecological models, PA behavior is affected by personal, socioeconomic, environmental, and policy factors [3]. Thus, to increase population-wide PA levels, national and local governments should focus on more structural changes at the environmental, policy, and societal levels [4]. In recent years, several large-scale, multi-city studies investigated the potential of the built environment to affect population-wide PA levels [5–7]. Based on recent systematic reviews, it can be concluded that changes in the built- and natural environments can lead to changes in PA levels of adults, and especially to changes in active transport (AT), i.e. walking and cycling for transport [8–11]. Moreover, AT might result in additional health benefits over vehicle-based transport, such as the reduction of the emission of, and exposure to, air pollution, and the improvement of neighborhoods’ livability by lowering the amount of motorized traffic [12, 13].

Although existing systematic reviews identified relationships between the environment and some types of physical activity behavior, the evidence remains inconclusive. One of the main issues is that the available evidence differs in measuring methods, quality and contexts. More longitudinal, context-specific research is needed to unravel the mechanisms that play a role in the relationship between the environment and behavior [14]. From previous research, we know that exposure to a new or redesigned infrastructure might increase the chance of engaging in AT [15]. However, exposure can be defined in various ways. Measures of exposure might be area-based, mostly consisting of administrative spatial boundaries, whereby exposure is treated as living in a specific area [16]. As exposure might vary within geographical areas, some studies use proximity (e.g. length or travel duration) as a continuous or ordinal measure of exposure [17, 18]. However, this approach assumes that the proximity of the home location to a specific environment is central to classify exposure to this environment [16]. Over the past decade, GPS-based approaches have increasingly been used to assess the actual exposure to a certain area, by combining GPS and geographical information systems (GIS). Following this trend, an increasing number of studies combine device-based location measurements with device-based PA measurements [19, 20]. This type of measurements prevents inconsistencies that typically occur when using self-reported PA measurements, such as inaccurate reporting and reporting bias [21]. However, large-scale evaluations exploring the effects of major infrastructural changes on the PA behavior of adults using both GPS and accelerometry are lacking.

In the city of Maastricht in the Netherlands, a highway crossing several deprived neighborhoods was tunneled in 2016 and the vacant space on top of this tunnel was prioritized for pedestrians and cyclists. The tunneling of this highway has led to a noise reduction of between 5 and 20 dB (depending on the exact location) and a decrease in the amount of nitrogen and particulate matter in the area [22]. Besides air quality, the tunneling also provided the opportunity to evaluate the effect on PA behavior. The aim of this study was to evaluate the effect of tunneling a highway on the overall PA and transport-based PA of people living in the vicinity of this major infrastructurally changed area, called The Green Carpet, compared to individuals living further away within the same city, and individuals living in another city in the same region. A second aim of this study was to explore the differences in PA for individuals that actually used or did not use the Green Carpet. This is the first large-scale evaluation to use individual-level device-based measurements of both PA and location in adults.

## Methods

### The project: Green Carpet

Since its opening in 1959, the highway A2 crosses residential areas in the east of the city of Maastricht. Due to the enormous increase in traffic over time, the burden on the residents of these areas also increased over time. Therefore, a double-layered tunnel was built to facilitate the traffic passing through the city (www.mijngroeneloper.nl/het-plan/information-english). To accommodate the remaining local traffic in the areas on top of the tunnel, two one-way streets were constructed. These one-way streets were separated by a semi-paved middle section, prioritized for use by pedestrians, cyclists and for recreation. This middle section was separated from the adjacent streets by wide strips of grass and trees, creating the so-called ‘Green Carpet’. The Green Carpet has a length of 2.3 kilometers. The semi-paved middle section has a width of about 6 meters, while the entire profile of the middle section, the strips with greenery and adjacent one-way streets is about 30 meters in width The Green Carpet was officially opened in spring 2018, but constructions of houses and facilities will continue up until 2026. Images of the intervention area before and after the opening of the Green Carpet can be found in the supplementary material (Figure S1 and S2). Details about the origin and context of the Green Carpet project have been described elsewhere [23].

### Study design and participants

This study used data from a non-randomized natural experiment. Natural experiments are alternatives to RCTs in cases in which it is practically or ethically impossible to manipulate exposure to an intervention, such as major changes in infrastructure [24]. The participants of this study were adult (≥18 years) inhabitants of Maastricht and Heerlen, two cities in the South-Limburg region of the Netherlands, which have about 120,000 and 100,000 inhabitants, respectively. Individuals who were not able to walk without walking aids or were not able to fill out a Dutch questionnaire were excluded from participation. Eligible participants were recruited via social media, posters, flyers at supermarkets and local events, advertisements in local and regional newspapers, and via personalized mailings to a random sample of the inhabitants.

Baseline measurements of the experiment were performed before the opening of the Green Carpet, between September 2016 and June 2017. The follow-up measurement was conducted between September 2018 and June 2019. Participants were measured in approximately the same week of the year at baseline and during the 2-year follow-up. On average, the time between the opening of the Green Carpet and the follow-up measurement of the individuals in Maastricht was 9.8 months (median: 10.5 months, range: 3-15 months).

The Maastricht University Medical Center (MUMC+) medical ethics committee reviewed the study protocol and concluded that formal ethical approval was not required (METC 16-4-109). All participants provided written informed consent. The study is registered at the Netherlands Trial Register (NL8108).

### Procedures

PA levels and location data were collected using device-based measurements, by the Actigraph GT3X+ activity monitor (Actigraph, Pensacola, FL, USA) and the Qstarz BT-Q1000XT GPS logger (Qstarz International Company, Taipei, Taiwan). Participants were instructed to wear both devices on an elastic belt on the right hip, for seven consecutive days at daytime only. Devices were removed during activities involving water, i.e. swimming and showering, and overnight, when the participant charged the GPS logger.

Raw accelerometry data (30 Hz) of the vertical axis were downloaded into Actilife version 6.11.7 (Actigraph, Pensacola, FL, USA) and converted to activity counts for 60-second epochs. GPS data were downloaded using Qtravel software version 1.52.000 (Qstarz International Company, Taipei, Taiwan) in epochs of 10 seconds. Accelerometry and GPS data were merged into 60-second epochs using the Human Activity Behavior Identification Tool and data Unification System (HABITUS), which is an updated version of the Personal Activity and Location Measurement System (PALMS) [25]. the GPS and accelerometry data were processed and filtered in HABITUS. Freedson’s cut points (1998) were applied to distinguish sedentary behavior (SB; <100 counts per minute) and light physical activity (LPA; >100 activity counts per minute) from moderate-to-vigorous physical activity (MVPA; >1952 counts per minute) [26].

Invalid GPS data points were identified based on extreme changes in speed (>130 km/hour) and elevation (1000m) between two epochs. Data points were distinguished as ‘stationary’ points, and points that were recorded during a trip. Activity was classified as a trip if the distance traveled was at least 100 meters and the duration exceeded 120 seconds. A stop of at least 120 seconds at one location was marked as a pause point and a pause of more than 180 seconds was marked as the endpoint of a trip. Periods of at least 60 minutes of zeros were classified as non-wear time and excluded from the analyses. The transport classification algorithm had a minute-level sensitivity of 88.5%, a specificity of 93.4%, and a positive predictive value of 74.9% [25]. The device-based measurements were considered valid if there were at least four days, regardless of week or weekend days, with a minimal wear time of 8 hours per day [27].

**Fig. 1:**
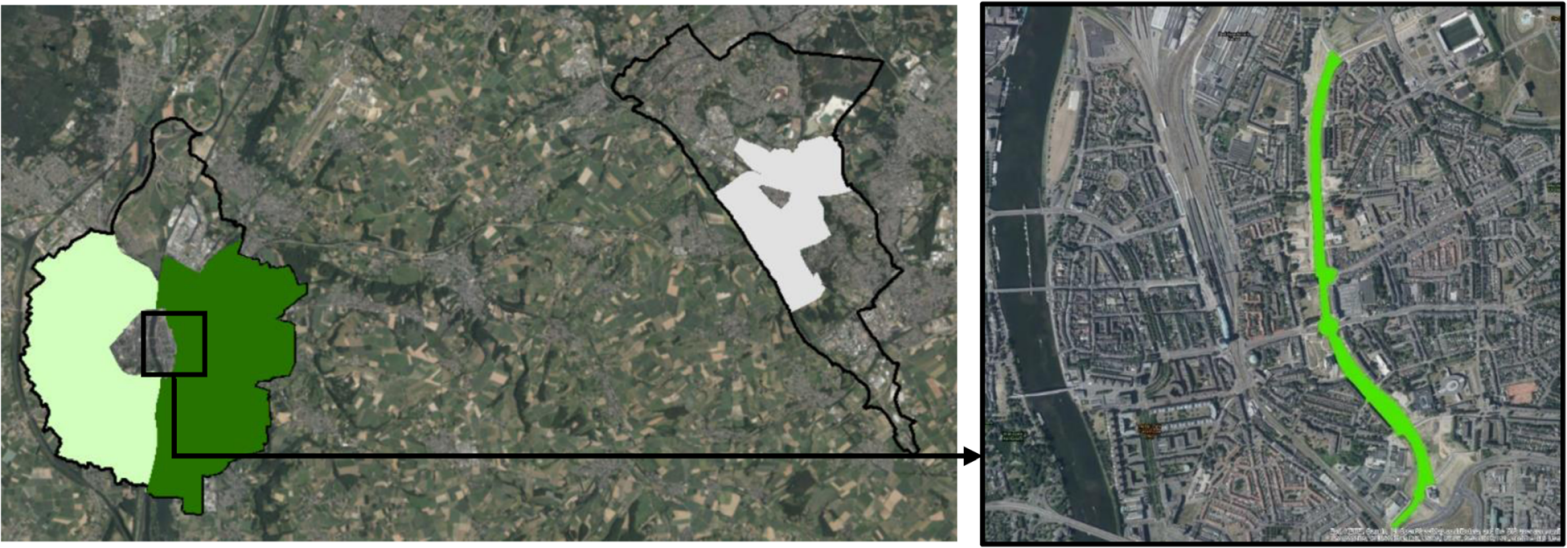
Area-based exposure groups and Green Carpet Area. Left: no exposure (white), minimal exposure (light green) and maximal exposure (dark green) areas in Maastricht and Heerlen. Right: 2.3 km Green Carpet on top of the A2 highway tunnel. Map: Source: Esri, DigitalGlobe, GeoEye, Earthstar Geographics, CNES/Airbus DS, USDA, USGS, AeroGRID, IGN, and the GIS User Community.

The HABITUS output was entered into a purpose-built PostgreSQL geodatabase which was used to assign datapoints to pre-defined contexts or domains. In this process, datapoints were hierarchically assigned and categorized as being in the home domain, the work domain, on the Green Carpet (Fig. 1) or in the transport domain. Outcomes in this study are the percentage of SB, LPA and MVPA of the total wear time, the percentage of transport-based SB, LPA and MVPA and the percentage of SB, LPA and MPVA at the Green Carpet.

### Measures of exposure

Participants belonged to one of the area-based exposure groups, based on the distance of their residential area to the Green Carpet area. The ‘maximal exposure’ group consisted of individuals that lived in the neighborhoods directly bordering the Green Carpet, situated at the East side of the city center (Fig. 1; Dark green; East Maastricht, South-East Maastricht). The expected exposure to the Green Carpet, was largest in this group. The ‘minimal exposure’ group consisted of inhabitants of Maastricht who lived on the western side of the river Meuse and outside the city center (Fig. 1; light green). Participants from these neighborhoods (West Maastricht, North-West Maastricht, South-West Maastricht), might visit the Green Carpet area, but are less likely to be exposed to this area. Individuals living in the ‘no exposure’ area were inhabitants of the city of Heerlen. Participants of this group were not expected to be exposed to the Green Carpet because they lived approximately 25 kilometers away from the Green Carpet. Heerlen was selected as comparison area because the selected neighborhoods in this city are comparable to Maastricht with regard to the number of inhabitants, urbanization and the geographical and cultural context.

Secondly, for all participants, the actual use of the Green Carpet area was determined using GPS data, whereby, for the participants of all three area-based groups, the use was defined and dichotomized to 0 (did not use Green Carpet area) and 1 (used Green Carpet area) at follow-up.

### Covariates

A questionnaire was distributed at baseline and follow-up to assess sociodemographic characteristics, including gender (0= male, 1= female), age, educational level (recoded into 0= lower educated, 1 = higher educated, for individuals with higher professional education or higher), work status (recoded into 0= not working, 1= working) and car ownership (recoded into 0= no car available in household, 1= one or more cars available in household). Also, health-related quality of life was assessed using the EQ-5D-3L questionnaire [28]. For each of the five domains of this questionnaire (mobility, self-care, daily activities, pain and mood) we created a dummy variable for individuals experiencing no problems (0) or any/severe problems (1) in a specific domain.

All study materials were distributed from local community centers, and after the 7-day data collection period ended, a member of the research team visited the participants at home to collect the materials.

### Statistical analyses

Descriptive statistics were used to describe and compare the baseline characteristics of the participants in the three groups of the experiment. To explore possible baseline differences and conduct dropout analyses, we performed T-tests and Chi-square tests on all covariates.

Normality was assumed based on the skewness and the kurtosis of the outcome measures. As mixed models are able to handle missing data in a longitudinal dataset when the covariates are present, changes in outcomes over time and intervention effects were determined using linear mixed models. Also, linear mixed models have the option to account for repeated measures within the individual. For each outcome, we first explored for each group the within-group changes by using time as a fixed factor in the model, while only accounting for repeated measures within persons. Next, an unadjusted model was created by adding an exposure group variable, accompanied by the interaction term between time x area-based exposure group. Lastly, a fully adjusted model was tested using the unadjusted model, supplemented with the covariates described above: age, gender, educational level, work status, car-ownership, and scores on EQ-5D. Sensitivity tests were conducted to validate the results with data of individuals that provided complete cases at both baseline and follow-up. Additionally, we further explored the differences in PA behavior between individuals that actually used the new infrastructure and individuals that did not, defined based on their GPS data. In these analyses, only individuals with valid data on both measurement moments were included. All statistical analyses were performed in SPSS version 24.0.0.2 (IBM Corp., Armonk, NY, USA) using a p-value of 0.05 as threshold for significance in all tests.

## Results

At baseline, 757 participants were recruited, of which 642 provided valid data at T0 and 362 provided valid data at both T0 and T1.

### Participants’ characteristics

At baseline, participants were on average 56.3 years old. The minimal exposure group was significantly older compared to the no exposure group (Table 1). About half of the sample were male and about half of the sample higher educated. Also, 54.8% of the participants were in employment, while the other 45.2% were retired or unemployed. Most of the participants had at least one car in their household.

**Table 1:** Baseline characteristics of the sample

	Total sample (***n***=642)	Maximal exposure (***n***=263)	Minimal exposure (***n***=179)	No exposure (***n***=200)
**Socio-demographics**				
Age (M (SD))	56.3 (16.1)	54.7 (16.2)	60.9 (13.4)^a^	54.2 (17.2)
Gender (% male)	46.2	42.4	47.5	50.0
Educational level (% higher educated)	52.5	55.6	48.0	52.5
Work status (% working)	54.8	57.3	47.7	57.7
Car ownership (% ≥ 1 car)	87.1	82.8	91.6	88.9
**Health-related quality of life**				
Mobility (% any or severe problems)	12.5	10.3	12.9	15.2
Self-care (% any or severe problems)	1.7	1.5	2.2	1.5
Daily activities (% any or severe problems)	11.1	11.1	9.0	13.1
Pain (% any or severe problems)	31.7	30.5	32.8	32.3
Mood (% any or severe problems)	11.5	10.7	12.4	11.8

*n* Sample size, *M* Mean, *SD* Standard deviation; ^a^ significantly different to the no exposure group at baseline

Dropout analyses on the participants’ characteristics showed some selective dropout at the no exposure group for educational level (χ^2^=8.325, *p*=.004). Also, in the minimal exposure and no exposure group, the percentage of individuals reporting any/severe problems regarding mood was higher in the group that dropped out at T1, compared to the longitudinal sample (minimal exposure group: χ^2^=5.031, *p*=.025, no exposure group: χ^2^=5.031, *p*=.040, respectively).

### Changes in total and transport-based PA – area-based exposure

At baseline, the average wear time ranged between 13.96 and 14.04 hours per day, and between 13.79 and 13.96 hours per day at follow-up (Table 2). The average number of wearing days ranged between 5.78 and 6.40 days at baseline and 5.64 and 6.34 at follow-up (data not shown). Within-group changes in wear time were not significant. In the maximal exposure group, the percentage of time spent in SB increased significantly between T0 and T1 (B=1.05, 95% CI 0.08; 2.01, *p*=.034), relating to 8.79 minutes per day. In contrast, for the minimal exposure group, the percentage of time spent in MVPA decreased significantly (B=-0.65, 95% CI -1.11; -0.20, *p*=.005). No changes were observed in the no exposure group. The wear time spent in transport ranged between 2.38 and 2.62 hours per day at baseline, and between 2.40 and 2.45 hours per day at follow-up. For the minimal exposure group, the wear time in transport decreased significantly (B=-0.18 95% CI 0.35; -0.01, *p*=.038). For the percentage of time in transport spent in SB, LPA and MVPA, a significant increase in SB and decrease in MVPA was found for the no exposure group (B=4.67, 95% CI 2.00; 7.34, *p*=.001, and B= -2.80, 95% CI -5.00; -0.60, *p*=.013, respectively). In absolute numbers, this relates to an average increase of 7.3 minutes per day of SB in transport, and an average decrease of 3.8 minutes per day of MVPA in transport. No changes were observed for the minimal and maximal exposure groups. Sensitivity analyses on the complete cases of this sample demonstrated similar trends (Supplementary material, Table S1).

**Table 2:** Observed (unadjusted) means with time as fixed factor and corrected for repeated measures in persons

Total PA		Maximal exposure				Minimal exposure				No exposure			
		*n*	*Mean (SE)*	*B (95% CI)*	*p*	*n*	*Mean (SE)*	*B (95% CI)*	*p*	*n*	*Mean (SE)*	*B (95% CI)*	*p*
Wear time (hrs/day)	T0	263	13.96 (0.09)			179	14.04 (0.10)			200	13.96 (0.11)		
	T1	154	13.96 (0.11)	-0.01 (-0.23; 0.21)	.955	111	13.86 (0.12)	-0.18 (-0.43; 0.07)	.157	97	13.79 (0.16)	-0.17 (-0.48; 0.14)	.279
% SB	T0	263	**63.63 (0.54)**			179	64.29 (0.65)			200	64.30 (0.58)		
	T1	154	**64.68 (0.61)**	**1.05 (0.08; 2.01)**	**.034**	111	64.79 (0.77)	0.49 (-0.93; 1.92)	.495	97	64.80 (0.82)	0.49 (-1.11; 2.10)	.544
% LPA	T0	263	32.03 (0.52)			179	31.56 (0.61)			200	31.21 (0.58)		
	T1	154	31.26 (0.58)	-0.77 (-1.79; 0.24)	.135	111	31.71 (0.73)	0.16 (-1.20; 1.52)	.818	97	30.78 (0.74)	-0.43 (-1.86; 0.99)	.548
% MVPA	T0	263	4.33 (0.19)			179	**4.15 (0.23)**			200	4.48 (0.20)		
	T1	154	4.03 (0.24)	-0.30 (-0.76; 0.16)	.198	111	**3.49 (0.24)**	**-0.65 (-1.11; -0.20)**	**.005**	97	4.47 (0.27)	-0.01 (-0.51; 0.48)	.964
Transport-based PA													
Wear time in transport (hrs/day)	T0	263	2.50 (0.06)			179	**2.62 (0.08)**			200	2.38 (0.07)		
	T1	154	2.45 (0.08)	-0.05 (-0.22; 0.12)	.572	111	**2.44 (0.08)**	**-0.18 (-0.35; -0.01)**	**.038**	97	2.40 (0.09)	0.02 (-0.14; 0.18)	.797
% SB	T0	263	48.01 (0.89)			179	49.62 (1.05)			200	**45.57 (1.04)**		
	T1	154	48.89 (1.11)	0.88 (-1.41; 3.17)	.448	111	50.00 (1.21)	0.38 (-2.08; 2.84)	.760	97	**50.24 (1.36)**	**4.67 (2.00; 7.34)**	**.001**
% LPA	T0	263	35.54 (0.73)			179	35.17 (0.89)			200	35.27 (0.84)		
	T1	154	35.57 (0.81)	0.03 (-1.71; 1.78)	.971	111	36.14 (1.02)	0.97 (-0.99; 2.93)	.330	97	33.31 (0.94)	-1.96 (-4.14; 0.21)	.077
% MVPA	T0	263	16.48 (0.79)			179	15.20 (0.92)			200	**19.16 (1.02)**		
	T1	154	15.49 (0.92)	-0.99 (-2.84; 0.86)	.251	111	13.92 (1.11)	-1.28 (-3.48; 0.92)	.293	97	**16.36 (1.12)**	**-2.80 (-5.00; -0.60)**	.**013**
PA at Green Carpet													
Wear time at Green Carpet (min/day)	T0	263	**2.67 (0.80)**			179	0.16 (0.93)			200	n.a.		
	T1	154	**3.22 (0.60)**	**2.65 (0.12; 5.19)**	**.040**	111	0.19 (0.75)	0.14 (-2.56; 2.85)	.918	97	n.a.	n.a.	n.a.
% SB	T0	263	47.73 (4.38)			179	**71.70 (7.06)**			200	n.a.		
	T1	154	37.30 (3.26)	-10.43 (-21.06; 0.21)	.055	111	**41.85 (7.22)**	**-29.85 (-49.57; -10.13)**	**.003**	97	n.a.	n.a.	n.a.
% LPA	T0	263	39.89 (3.76)			179	**16.14 (6.09)**			200	n.a.		
	T1	154	40.59 (3.24)	0.70 (-8.43; 9.83)	.880	111	**50.15 (7.15)**	**34.01 (16.16; 51.86)**	**.000**	97	n.a.	n.a.	n.a.
% MVPA	T0	263	**12.29 (3.09)**			179	12.63 (4.99)			200	n.a.		
	T1	154	**21.09 (2.68)**	**8.80 (1.18; 16.41)**	**.024**	111	8.65 (5.91)	-3.99 (-18.79; 10.81)	.596	97	n.a.	n.a.	n.a.

*PA* Physical activity, *SB* Sedentary behavior, *LPA* Light physical activity, *MVPA* Moderate-to-vigorous physical activity, *n* Sample size, *SE* Standard error, *B* Beta coefficient, *95% CI* 95% confidence interval, *n.a*. Not applicable, not enough cases to perform analyses

Average wear time spent on the Green Carpet increased significantly in the maximal exposure group from 2.67 minutes per day at baseline to 3.22 minutes per day at follow-up. The percentage of time spent in MVPA increased significantly from 12.29% at baseline to 21.09% at follow-up (B=8.80, 95% CI 1.18; 16.14, *p*=.024). The percentage of wear time at the Green Carpet spent in SB decreased, but this change was not significant. For the minimal exposure group, the average wear time spent on the Green Carpet was less than one minute and did not change over time. The percentage SB at the Green Carpet significantly decreased with 30%, from 71.07% to 41.85% (B=-29.85, 95% CI -49.57; -10.13, *p*=.003), and the amount of LPA increased with 34.01% (B=34.01, 95% CI 16.16; 51.86, *p*<.001).

**Fig. 2:**
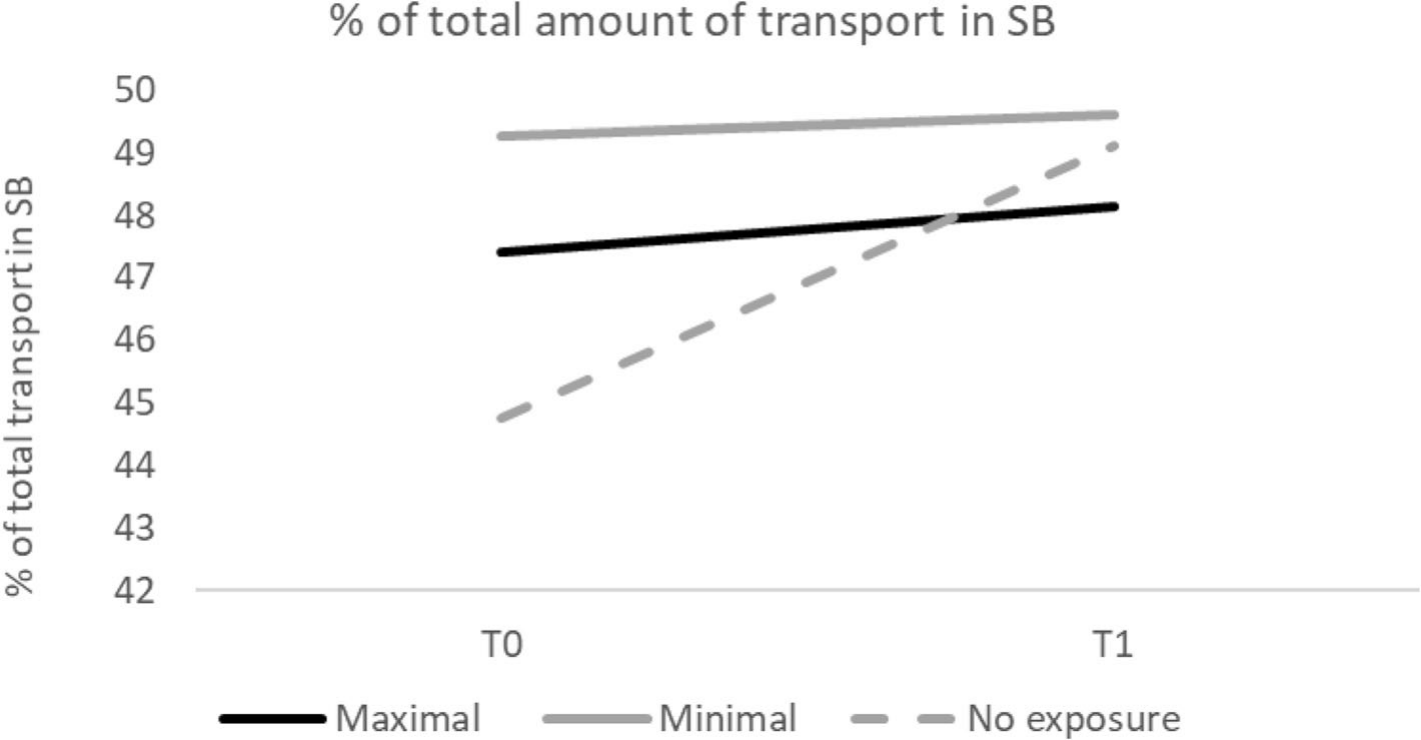
Visual representation of the time x exposure group interaction for the transport-based SB outcome.

Interactions between time x exposure group were determined to explore whether the changes over time were different for the three exposure groups (Table 3). In both the adjusted and unadjusted models, the change in transport-based SB was significantly different for the control group, compared to the maximal exposure group (B=-3.59, 95% CI -7.15; -0.02, *p*=.049) and minimal exposure group (B= -4.02, 95% CI -7.85, -0.19, *p*=.040) (Fig. 2). No significant interactions were found for the other total and transport-based PA outcomes.

**Table 3:** Estimates of time x exposure group in unadjusted and maximal adjusted linear mixed effects models

Total PA	Unadjusted model				Adjusted model^a^			
	Maximal vs. No exposure		Minimal vs. No exposure		Maximal vs. No exposure		Minimal vs. No exposure	
	*B (95% CI)*	*p*	*B (95% CI)*	*p*	*B (95% CI)*	*p*	*B (95% CI)*	*p*
% SB	0.68 (-1.13; 2.49)	.457	0.10 (-1.84; 2.04)	.920	0.92 (-0.92; 2.75)	.326	0.24 (-1.74; 2.22)	.809
% LPA	-0.38 (-2.12; 1.36)	.667	0.54 (-1.32; 2.41)	.567	-0.64 (-2.41; 1.12)	.472	0.44 (-1.46; 2.34)	.647
% MVPA	-0.27 (-0.94; 0.40)	.427	-0.62 (-1.34; 0.09)	.088	-0.28 (-0.96; 0.40)	.423	-0.69 (-1.43; 0.04)	.065
Transport-based PA								
% SB	**-3.82 (-7.29; -0.35)**	**.031**	**-4.24 (-7.96; -0.53)**	**.025**	**-3.59 (-7.15; -0.02)**	**.049**	**-4.02 (-7.85; -0.19)**	**.040**
% LPA	1.96 (-0.77; 4.70)	.159	2.75 (-0.18; 5.69)	.066	1.69 (-1.12; 4.49)	.238	2.91 (-0.11; 5.93)	.058
% MVPA	1.76 (-1.15; 4.66)	.236	1.50 (-1.62; 4.61)	.345	1.81 (-1.17; 4.79)	.234	1.15 (-2.06; 4.35)	.482

^a^ adjusted for age, gender, educational level, work status, car ownership and health-related quality of life; *PA* Physical activity, *SB* Sedentary behavior, *LPA* Light physical activity, *MVPA* Moderate-to-vigorous physical activity, *B* Beta coefficient, *95% CI* 95% Confident interval

### Changes in total and transport-based PA – actual users

When focusing on individuals that actually used the Green Carpet versus individuals that did not use the Green Carpet, we found a significant decrease in wear time over time in both groups (Table 4). In both groups, no significant changes were found in the total percentage of SB, LPA and MVPA. Also, the total amount of time spent in transport remained the same in both groups, and the percentage transport-based SB and LPA did not significantly change over time. For the group that did not use the Green Carpet area, the total percentage of transport-based MVPA decreased significantly (B=-1.78, 95% CI -3.43; -0.12, *p*=.035) (5.70 minutes/day) while the percentage of MVPA remained stable in the group of individuals that actually used the Green Carpet. No significant changes were found in the wear time or physical activity levels on the Green Carpet.

**Table 4:** Observed means with time as fixed factor and corrected for repeated measures in persons, comparison of users and non-users the Green Carpet area

Total PA		Users (*n*=108)			Non-users (*n*=208)		
		*Mean (SE)*	*B (95% CI)*	*p*	*Mean (SE)*	*B (95% CI)*	*p*
Wear time (hrs)	T0	**14.35 (0.10)**			**14.14 (0.10)**		
	T1	**14.11 (0.12)**	**-0.24 (-0.47; -0.01)**	**.040**	**13.87 (0.10)**	**-0.27 (-0.49; -0.06)**	**.011**
% SB	T0	63.32 (0.78)			65.32 (0.55)		
	T1	64.44 (0.81)	1.11 (-0.00; 2.22)	.050	65.37 (0.60)	0.050 (-1.00; 1.10)	.925
% LPA	T0	30.73 (0.53)			32.25 (0.73)		
	T1	30.96 (0.56)	-0.85 (-1.95; 0.25)	.127	31.40 (0.76)	0.23 (-0.75; 1.21)	.644
% MVPA	T0	4.43 (0.27)			3.95 (0.21)		
	T1	4.17 (0.25)	-0.26 (-0.70; 0.18)	.241	3.67 (0.20)	-0.28 (-0.62; 0.059)	.105
Transport-based PA							
Wear time (hrs)	T0	2.63 (0.08)			2.47 (0.07)		
	T1	2.55 (0.08)	-0.09 (-0.27; 0.10)	.347	2.42 (0.07)	-0.06 (-0.17; 0.06)	.343
% SB	T0	48.21 (1.42)			49.14 (1.00)		
	T1	48.55 (1.34)	0.34 (-2.32; 3.00)	.802	50.72 (0.99)	1.58 (-0.38; 3.53)	.114
% LPA	T0	35.14 (1.08)			34.63 (0.84)		
	T1	35.34 (1.01)	0.20 (-1.82; 2.22)	.847	34.83 (0.75)	0.20 (-1.42; 1.82)	.808
% MVPA	T0	16.65 (1.19)			**16.23 (0.94)**		
	T1	16.12 (1.16)	-0.53 (-2.52; 1.45)	.596	**14.46 (0.86)**	**-1.78 (-3.43; -0.12)**	**.035**
PA at Green Carpet							
Wear time (min)	T0	2.50 (0.94)			n.a.		
	T1	2.23 (0.45)	-0.27 (-1.53; 2.07)	.767	n.a.	n.a.	n.a.
% SB	T0	44.80 (5.11)			n.a.		
	T1	37.83 (3.96)	-6.97 (-19.32; 5.39)	.266	n.a.	n.a.	n.a.
% LPA	T0	43.93 (4.42)			n.a.		
	T1	42.93 (3.93)	-1.00 (-11.61 ; 9.60)	.851	n.a.	n.a.	n.a.
% MVPA	T0	11.22 (3.49)			n.a.		
	T1	18.99 (3.29)	7.77 (-0.85; 16.39)	.077	n.a.	n.a.	n.a.

*PA* Physical activity, *SB* Sedentary behavior, *LPA* Light physical activity, *MVPA* Moderate-to-vigorous physical activity, *SE* Standard error, *B* Beta coefficient, *95% CI* 95% confidence interval, *n.a.* Not applicable, not enough cases to perform analyses

In both the adjusted and unadjusted models, the interactions between time x exposure were not significant for any of the PA outcomes (Table 5).

**Table 5:** Estimates of time x exposure group in unadjusted and maximal adjusted linear mixed effects models

Total PA	Unadjusted model		Adjusted model*	
	Users vs. Non-users		Users vs. Non-users	
	*B (95% CI)*	*p*	*B (95% CI)*	*p*
% SB	1.06 (-0.59; 2.72)	.206	1.19 (-0.51; 2.89)	.169
% LPA	-1.08 (-2.66; 0.49)	.176	-1.24 (-2.86; 0.37)	.130
% MVPA	0.02 (-0.55; 0.58)	.948	0.05 (-0.53; 0.63)	.865
Transport-based PA				
% SB	-1.24 (-4.55; 2.07)	.462	-0.76 (-4.20; 2.68)	.664
% LPA	-0.00 (-2.67; 2.66)	.998	-0.49 (-3.26; 2.27)	.726
% MVPA	1.24 (-1.45; 3.94)	.365	1.25 (-1.54; 4.05)	.379

*adjusted for age, gender, educational level, work status, car ownership and health-related quality of life; *PA* Physical activity, *SB* Sedentary behavior, *LPA* Light physical activity, *MVPA* Moderate-to-vigorous physical activity, *B* Beta coefficient, *95% CI* 95% confident interval

## Discussion

The aim of this study was to evaluate changes in overall and transport-based PA and SB of people living near an area of major infrastructural change and to compare it with transport-based PA changes in individuals living further away. In addition, we evaluated the differences in total PA outcomes for individuals who were actually using the new infrastructure and those who were not.

For the total PA levels, we found a decrease in the percentage MVPA in the minimal exposure group (5.9%, 5.94 minutes/day) and an increase in the percentage of SB (0.3%, 8.8 minutes/day) in the maximal exposure group. The PA levels in the control group did not significantly change over time. For both the adjusted and unadjusted models, the trends in total PA over time did not differ across the three groups. Some previous studies also found decreased levels of MVPA across study groups, at short-term follow-ups [17, 29]. As the sample in the minimal exposure group was significantly older compared to the others, the decrease in the unadjusted MVPA levels might be an age-related decline [30].

For transport-based PA levels, we found a decrease in the percentage MVPA (2.8%; -3.8 minutes/day) and an increase in the percentage SB for the no exposure group (4.7%; 7.3 minutes/day), while for the maximal and minimal exposure group the levels of transport-based SB and MVPA did not change over time. However, it should be noted that baseline levels of transport-based MVPA were higher in the no exposure group compared to the other areas. The decrease might be an adaptation to MVPA levels that are more comparable to the average. Also, although the participants in the no exposure group were not exposed to the Green Carpet, some smaller scale environmental changes have been going on in Heerlen. During this first follow-up measurement, the main railway station and its surroundings were under construction, and the parking costs of some parking spaces in the city center were reduced to make visiting the inner city more attractive. However, it is unclear if and to what extent this impacted on the study results. Longer-term follow-ups are necessary to see if this trend continues.

Although the total and transport-based PA levels did not increase over time, we found an 8% increase of MVPA on the Green Carpet. This implies that when participants were on the Green Carpet, they more often were moderate-to-vigorously active. As the Green Carpet was only a transport route at the time of follow-up, this increase in MVPA and the increase of time spent in this area probably relates to an increase in brisk walking or cycling. For the minimal exposure group, we found a decrease in SB and increase of LPA on the Green Carpet. Given the distance to the Green Carpet and the PA levels of the participants of this group, this change might indicate that the Green Carpet led to relatively less car use and more light-active forms of PA, such as cycling. Hereby, the Green Carpet might act as a route for active trips that were previously made using a car or public transport. These results indicate that the Green Carpet evokes behavioral changes at the Green Carpet, but this did not yet lead to additional PA.

Lastly, we compared users and non-users of the Green Carpet area. For individuals that used the Green Carpet, no changes were observed in transport-based PA, while in the non-user group, transport-based MVPA decreased by 1.8%. Although this difference is slightly smaller compared to the changes in area-based exposure groups, the trends over time were comparable. This means that, possibly, the changes between the area-based groups might reflect the differences between visiting and non-visiting/using individuals. This would imply that living in a Green Carpet area prevented a decrease in transport-based MVPA only for actual users. However, even though we adjusted for several covariates, more in-depth analyses are needed to reduce the possible influence of selective daily mobility bias in the use of the Green Carpet [31]. Previous research showed that users of new infrastructures might be the more active individuals [32], but our results did not suggest that this was an issue in our study. Further, the *time x exposure* interaction was not significant. Thus, although there was a significant decrease in transport-based PA in the non-visiting group, the trend over time did not differ between users and non-users of the Green Carpet. Moreover, the changes in PA levels at the Green Carpet were of a same magnitude compared to the area-based exposure groups, but were not statistically significant. Remarkably, the average wear time while in the Green Carpet showed an opposite trend in the user groups, compared to the area-based study groups. Probably, this is due to the increased connectivity of the area and the removal of traffic lights that caused major traffic jams.

Although we did not find increases in transport-based PA, we found that individuals in the exposed areas, on average, did not decrease the amount of transport-based MVPA, in contrast to the control area. In two systematic reviews it was argued that, in general, studies were able to detect positive behavioral changes when the follow-up measurement took place at least 6-12 months after the opening of the new infrastructure [8, 11]. In this study, the average time between the opening of the Green Carpet and the follow-up measurement was 9.8 months, with a median of 10.5 months. Therefore, more follow-up measurements are necessary to investigate the longer-term effects of this infrastructural change on PA behavior. Since the construction of dwellings and facilities is still ongoing until 2026 and the planted trees need time to grow to become a more attractive place for leisure time PA, longer-term assessments are warranted.

In the current study, we focused on the general effects of an infrastructural change to the built environment on PA, whereby we adjusted for several covariates, but did not consider possible subgroup effects. As proposed by theoretical models, individual-level socioeconomic, cultural and demographic characteristics might moderate the effect of the environment on PA [33]. Also, individuals’ perceptions of the environment might mediate this relationship between environment and behavior, but this was not taken into account in this study [34]. Additional analyses are needed to further investigate the effects of individual-level moderating and mediating factors.

Strengths of this study are its longitudinal character, large-scale device-based measurements, and the inclusion of sub analyses on users and nonusers of the Green Carpet, next to area-based exposure measures. To our knowledge, this is the first study on this scale that uses both GPS and accelerometers in a longitudinal approach to investigate effects of an infrastructural project on PA behavior, which improves the validity and reliability of studies into the relationship between environment and behavior.

An important limitation of this study is the possible misclassification of the datapoints that were classified as ‘in transport’. Giving the positive predictive value of 74.9%, the algorithm is slightly susceptible to false positives. This means that some of the 60-second time periods might be classified as trips, while they are not [25]. Another limitation of this study is the dropout of participants between baseline and follow-up measurements, due to several reasons. The persons that dropped out spent slightly more time in MVPA at baseline. As these models provide the opportunity to handle missing outcome data based on valid covariates on baseline, these data emphasized the importance of using linear mixed models. However, sensitivity analyses showed that the findings were similar for the sample that contained only complete baseline and follow-up measurements. Also, the percentage of people that were lower educated and experiencing problems regarding mood was significantly higher in the dropout group, compared to the longitudinal sample. Thus, sensitivity analyses did not reveal significant differences in the outcome measures between the dropout group and longitudinal sample, nor between lower and higher educated individuals and people with or without problems regarding mood.

Further, when interpreting the results of this study, the relative nature of the data should be noted. The average weartime of the devices was about 14 hours per day, whereby consequently about 10 hours of the day were not recorded. Although a significant part of these hours is expected to be sleep time, these hours partially consist of non-weartime during the day. In both cases, we did not correct for this in the current analyses. Also, as a day consists of 24 hours, an increase in the total time in one behavior (SB, LPA or MVPA) causes a decrease in the total time spent in on or more of the other domains [35]. Compositional data analyses (CoDa) accounts for this codependency by handling a ‘time budget’ of 24 hours per day, of which time is allocated to specific behaviors or physical activity domains. Previous research has shown how this type of analyses might help to further understand patterns of physical activity behaviors during the day [36], or examine the combined effects of sleep, SB, LPA and MVPA on health outcomes [37]. Hereby, CoDa provides opportunities for future research. Lastly, the recruited group was older and higher educated than the total population in the selected areas. Despite controlling for these covariates in the statistical models, results might be less generalizable to a younger and lower educated sample.

## Conclusion

This study showed that tunneling a highway passing through residential areas of Maastricht city, and reconstructing the new open space in favor of non-motorized and slow traffic did not significantly increase total or transport-based PA, within a year after opening in 2018. However, the amount of transport-based MVPA showed a stable trend over time in the exposure groups, in contrast to the control group. The percentage MVPA at the Green Carpet area increased significantly for individuals from the maximal exposure group. For the minimal exposure group, the percentage of time spent in SB when being at the Green Carpet decreased, while LPA increased significantly. This implies that the PA patterns within the Green Carpet area changed over time, but did not yet lead to an increase in the total volume of PA. Although the results differed between the area-based exposure and individual-level exposure analyses, the trends were similar for both analyses. This suggests that area-based differences might reflect the differences between users and nonusers of the Green Carpet. Due to possible interference of selective mobility bias, however, the results should be interpreted carefully. Further, this paper reflects that the relationship between infrastructure and PA is not unambiguous, as it depends on the context, and thereby interacts with the contextual factors in the larger ecosystem. Finally, to investigate longer-term effects, more research is needed.

## Supplementary Information

**Additional file 1.**

**Additional file 2.**

**Additional file 3.**

## Data Availability

The datasets generated during and/or analyzed during the current study are available from the corresponding author on reasonable request.

## Abbreviations

PA: Physical activity
AT: Active transport
GPS: Global positioning systems
GIS: Geographical information system
RCTs: Randomized controlled trials
HABITUS: Human Activity Behavior Identification Tool and data Unification System
PALMS: Personal Activity and Location Measurement System
SB: Sedentary behavior
LPA: Light physical activity
MVPA: Moderate-to-vigorous physical activity
