# Supplementary material for: Tunneling a crosstown highway: a natural experiment testing the longitudinal effect on physical activity and active transport
Source: Int J Behav Nutr Phys Act. 2021 Aug 26;18:111. doi: 10.1186/s12966-021-01180-1 (PMC8390260; doi:10.1186/s12966-021-01180-1)
Supplement: Supplementary file 1 — Additional file 1. [file 12966_2021_1180_MOESM1_ESM.docx]

**Sample recruitment**

Eligible participants were recruited via social media, posters, flyers at supermarkets and local events, advertisements in local and regional newspapers, and via personalized mailings to a random sample of the inhabitants. This broad recruitment strategy allowed us to recruit individuals from different age groups, and individuals with and without access to the internet.

**Representativeness of recruited group from target group**

The mean age of the recruited sample was higher than the mean age of the (adult) population of the target group. On baseline, the mean age in the no exposure group was 54.2 while the mean age of adults in the target group was 50.1. For the minimal exposure group, this was 60.9 vs. 49.7, and for the maximal exposure group 54.7 vs. 47.1, respectively. In terms of gender, the recruited group is similar to the target group for the no exposure group (50% male in the recruited sample vs. 50% male in the target population) and minimal exposure group (47.5% male in the recruited sample vs. 47.3% male in the target population). In the maximal exposure group, 42.4% of the recruited group was male, relative to 48.1% in the target population. Also, about 46% of the recruited sample was higher educated, while the percentage of higher educated individuals in the target population is about 30%. These variables were added as covariates to the final statistical models and the consequences for generalizability are discussed in the discussion paragraph.

**How analyzed sample differed from recruited sample**

At baseline, 757 participants were recruited, of which 642 provided valid data at baseline. In total, 37 participants were excluded for analyses because they did not provide a valid questionnaire. Other dropouts at baseline did not provided valid accelerometry or GPS data. On average, the analyzed sample was 57.3 year old, compared to 56.4 years of the total recruited sample (that provided a valid questionnaire to provide age). Of the analyzed sample, 46.2% was male, compared to 46.5% in the recruited sample. Regarding educational level, 52.5% was higher educated in the analyzed sample, compared to 48.5% in the recruited sample. In summary, the analyzed sample was similar to the recruited sample regarding age and gender. For educational level, the percentage of higher educated individuals was slightly higher in the analyzed sample compared to the recruited sample.

**How missing data was handled**

Participants who had missing data for both the questionnaire and device-based measurements were excluded for the analysis. To account for missing data in the follow-up, a linear mixed model was used. Linear mixed models are able to handle missing data when the covariates (at baseline) are present. Two steps were taken: we performed drop-out analysis to explore the characteristics of the participants that dropped out between T0 and T1. There were some differences between those two groups, which emphasized the importance of adding covariates into the models to account for this. Second, we performed sensitivity analyses on the group that provided data on both measurement moments. These models showed trends that were similar to the main analyses.
