# Supplementary material for: Tunneling a crosstown highway: a natural experiment testing the longitudinal effect on physical activity and active transport
Source: Int J Behav Nutr Phys Act. 2021 Aug 26;18:111. doi: 10.1186/s12966-021-01180-1 (PMC8390260; doi:10.1186/s12966-021-01180-1)
Supplement: Supplementary file 2 — Additional file 2. [file 12966_2021_1180_MOESM2_ESM.docx]

# Sensitivity analyses on longitudinal data

Table S1 Observed means with time as fixed factor and corrected for repeated measures in persons – longitudinal data only.

|  |  | Maximal exposure (N=154) | | | Minimal exposure (N=111) | | | No exposure (N=97) | | |
| --- | --- | --- | --- | --- | --- | --- | --- | --- | --- | --- |
| Total physical activity | | *Mean (SE)* | *B (95% CI)* | *p* | *Mean (SE)* | *B (95% CI)* | *p* | *Mean (SE)* | *B (95% CI)* | *p* |
| % SB | T0 | 63.78 (0.68) |  |  | 64.97 (0.83) |  |  | 65.11 (0.75) |  |  |
|  | T1 | **64.90 (0.70)** | **1.13 (0.12; 2.13)** | **.028** | 65.08 (0.67) | 0.10 (-1.40; 1.61) | .894 | 65.08 (0.88) | -0.03 (-1.72; 1.66) | .970 |
| % LPA | T0 | 31.93 (0.64) |  |  | 31.05 (0.78) |  |  | 30.82 (0.73) |  |  |
|  | T1 | 31.18 (0.65) | -0.74 (-1.78; 0.29) | .157 | 31.51 (0.82) | 0.46 (-0.97; 1.90) | .525 | 30.67 (0.80) | -0.14 (-1.64; 1.35) | .850 |
| % MVPA | T0 | 4.29 (0.24) |  |  | 3.97 (0.29) |  |  | 4.07 (0.31) |  |  |
|  | T1 | 3.91 (0.24) | -0.38 (-0.77; 0.14) | .059 | **3.42 (0.27)** | **-0.55 (-1.04; -0.07)** | **.025** | 4.25 (0.32) | 0.18 (-0.35; 0.70) | .507 |
| Transport domain | |  |  |  |  |  |  |  |  |  |
| % SB | T0 | 49.23 (1.23) |  |  | 50.49 (1.36) |  |  | 46.26 (1.56) |  |  |
|  | T1 | 49.60 (1.22) | 0.37 (-2.12; 2.86) | .770 | 49.95 (1.34) | -0.54 (-3.22; 2.15) | .693 | **50.38 (1.53)** | **4.11 (1.17; 7.06)** | **.007** |
| % LPA | T0 | 34.53 (0.94) |  |  | 34.64 (1.16) |  |  | 35.15 (1.24) |  |  |
|  | T1 | 35.19 (0.87) | 0.66 (-1.24; 2.57) | .494 | 35.96 (1.14) | 1.31 (-0.80; 3.43) | .222 | 33.37 (1.02) | -1.78 (-4.33; 0.76) | .167 |
| % MVPA | T0 | 16.27 (1.03) |  |  | 14.87 (1.24) |  |  | 18.63 (1.55) |  |  |
|  | T1 | 15.23 (1.01) | -1.05 (-2.91; 0.82) | .270 | 14.07 (1.26) | -0.80 (-3.23; 1.63) | .516 | 16.22 (1.31) | -2.40 (-4.87; 0.06) | .056 |

PA= physical activity; SB= sedentary behavior; LPA= light physical activity; MVPA= moderate-to-vigorous physical activity; n= sample size; SE= standard error; B= beta coefficient; 95% CI= 95% confidence interval.

Pictures of the intervention area before and after the opening of the Green Carpet


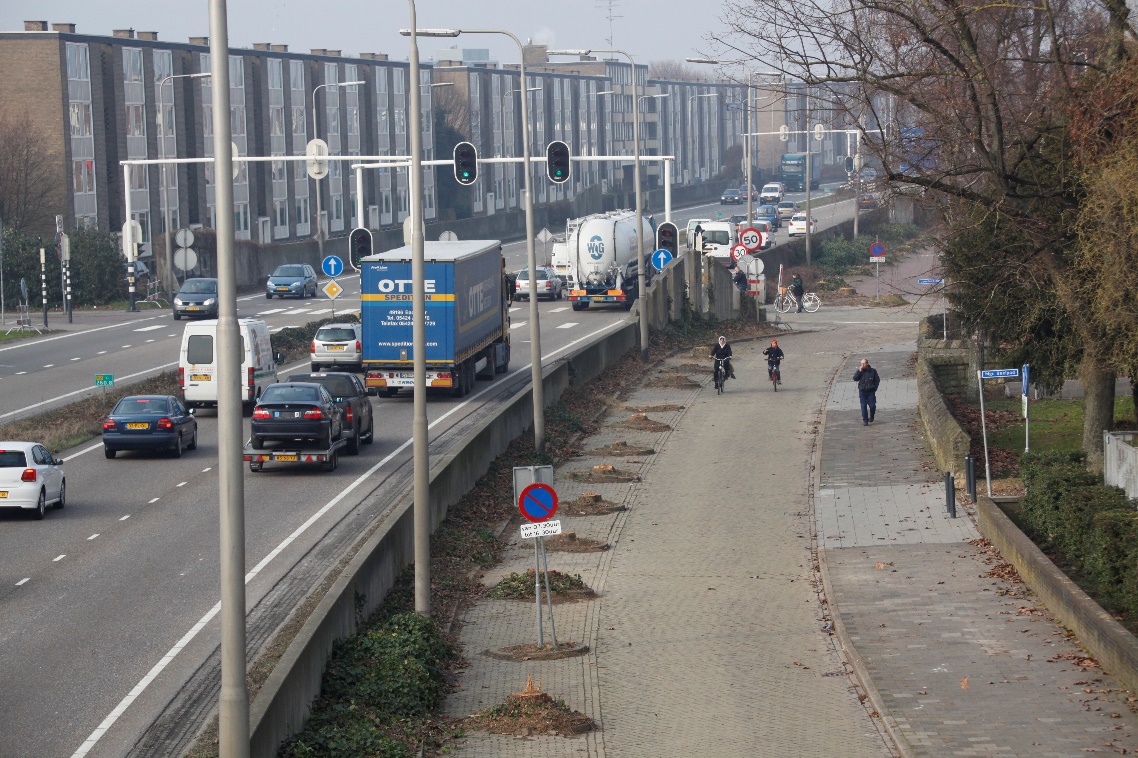


Figure S1. Picture of the intervention area before the highway A2 crossing the city of Maastricht before the opening of the tunnel and the Green Carpet. Source: Projectbureau A2 Maastricht


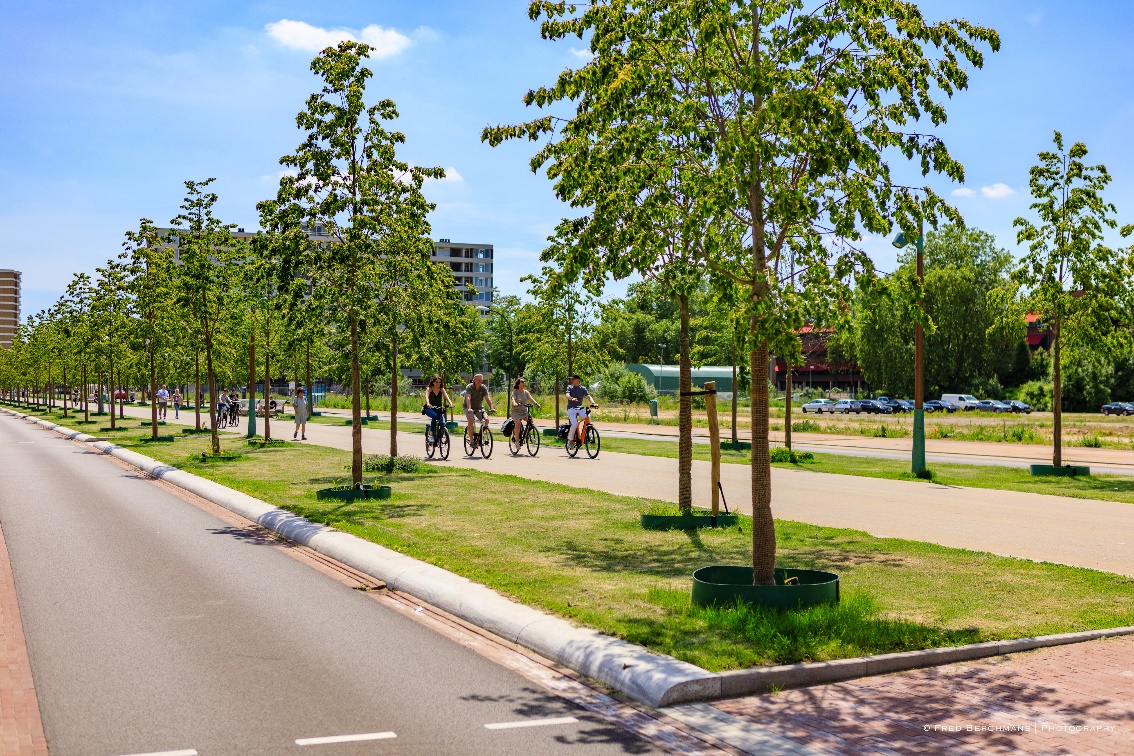


Figure S2. Picture of the Green Carpet showing the profile consisting of a semi-paved middle path, adjacent one-way streets, and green strips separating them. Source: Projectbureau A2 Maastricht/ Fred Berghmans.
