# Supplementary material for: Tunneling a crosstown highway: a natural experiment testing the longitudinal effect on physical activity and active transport
Source: Int J Behav Nutr Phys Act. 2021 Aug 26;18:111. doi: 10.1186/s12966-021-01180-1 (PMC8390260; doi:10.1186/s12966-021-01180-1)
Supplement: Supplementary file 3 [file 12966_2021_1180_MOESM3_ESM.docx]

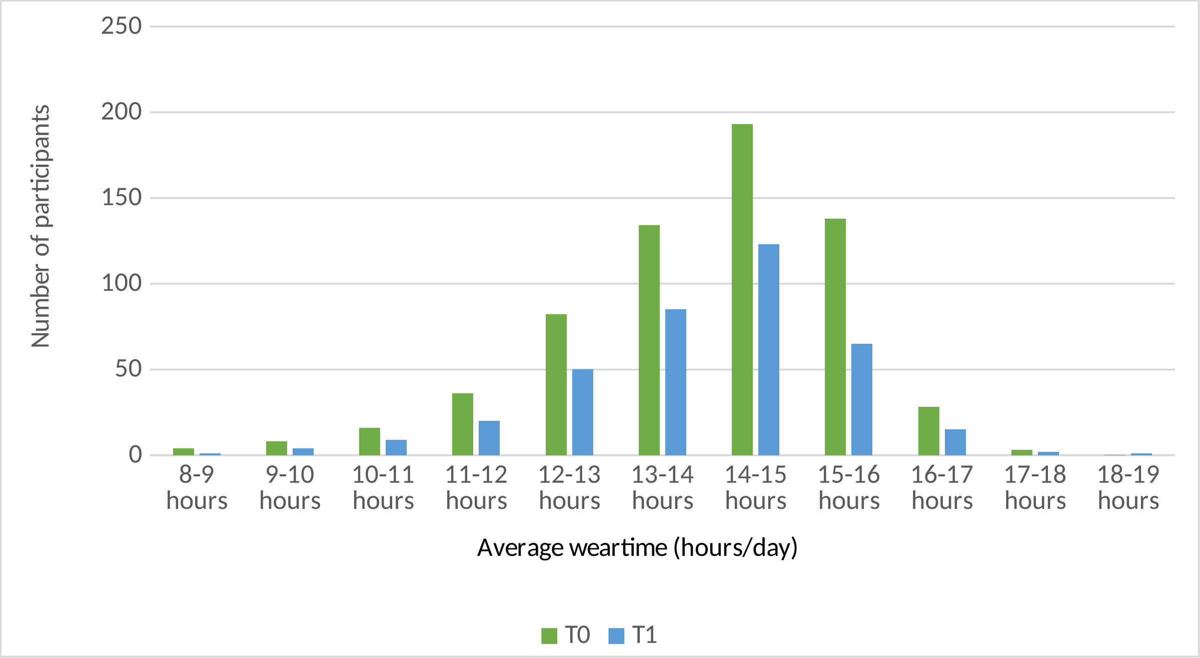
250
200
150
100
50
0
Number of participants
8-9 hours
9-10 hours
10-11 hours
11-12 hours
12-13 hours
13-14 hours
14-15 hours
15-16 hours
16-17 hours
17-18 hours
18-19 hours
Average weartime (hours/day)
T0
T1
